# CircuitsDB: a database of mixed microRNA/transcription factor feed-forward regulatory circuits in human and mouse

**DOI:** 10.1186/1471-2105-11-435

**Published:** 2010-08-23

**Authors:** Olivier Friard, Angela Re, Daniela Taverna, Michele De Bortoli, Davide Corá

**Affiliations:** 1 Center for Molecular Systems Biology, University of Torino, Via Accademia Albertina, 13 - I-10123 Torino, Italy; 2 CIBIO-Centre for Integrative Biology, University of Trento, Via delle Regole 101, I-38100 Trento, Italy; 3 Department of Oncological Sciences, c/o Institute for Cancer Research and Treatment (IRCC), School of Medicine, University of Torino, Str. Prov. 142 Km. 3.95, I-10060 Candiolo, Italy; 4 Molecular Biotechnology Center, University of Torino, Via Nizza 52, I-10126 Torino, Italy; 5 Systems Biology Lab, Institute for Cancer Research and Treatment (IRCC), School of Medicine, University of Torino, Str. Prov. 142 Km. 3.95, I-10060 Candiolo, Italy

## Abstract

**Background:**

Transcription Factors (TFs) and microRNAs (miRNAs) are key players for gene expression regulation in higher eukaryotes. In the last years, a large amount of bioinformatic studies were devoted to the elucidation of transcriptional and post-transcriptional (mostly miRNA-mediated) regulatory interactions, but little is known about the interplay between them.

**Description:**

Here we describe a dynamic web-accessible database, CircuitsDB, supporting a genome-wide transcriptional and post-transcriptional regulatory network integration, for the human and mouse genomes, based on a bioinformatic sequence-analysis approach. In particular, CircuitsDB is currently focused on the study of mixed miRNA/TF Feed-Forward regulatory Loops (FFLs), i.e. elementary circuits in which a master TF regulates an miRNA and together with it a set of Joint Target protein-coding genes. The database was constructed using an ab-initio oligo analysis procedure for the identification of the transcriptional and post-transcriptional interactions. Several external sources of information were then pooled together to obtain the functional annotation of the proposed interactions. Results for human and mouse genomes are presented in an integrated web tool, that allows users to explore the circuits, investigate their sequence and functional properties and thus suggest possible biological experiments.

**Conclusions:**

We present CircuitsDB, a web-server devoted to the study of human and mouse mixed miRNA/TF Feed-Forward regulatory circuits, freely available at: http://biocluster.di.unito.it/circuits/

## Background

Gene regulation is one of the most important molecular mechanisms occurring in a eukaryotic cell or organism. Control of gene expression is crucial for normal development and maintenance of healthy cells, and alterations from standard coordination programs can lead to severe diseases including cancer. The numerous events going from a DNA gene sequence to the corresponding protein are carefully controlled: from the control of transcription initiation to post-translational modifications that ultimately indicate the fate of the protein product. The primary regulation of gene expression is thought to be performed by Transcription Factors (TFs), proteins that are able to positively or negatively coordinate gene transcription through the interaction with specific recognition DNA motifs usually located in the gene promoter regions (see [1,2] for recent reviews and perspectives).

In the last years, however, an additional class of gene regulators emerged: the microRNAs (miRNAs). miRNAs are short (~22nt) endogenous non-coding RNAs able to negatively regulate gene expression at the post-transcriptional level, via mRNA cleavage or translational repression. To this purpose, antisense complementary base-pair matching between a mature miRNA and its specific target sequences, located in the 3'-UTR of the regulated mRNAs, is usually required (reviewed e.g. in [3]).

As a consequence of the above mentioned discoveries, the study of gene regulation has undergone a deep change of perspective. While past studies usually dealt with individual regulatory interactions, it has become clear that the only way to understand the regulatory activity of a eukaryotic genome is to directly address the complex, combinatorial nature of the whole ensemble of DNA *cis *and *trans *elements involved in such a process. Despite numerous efforts, mechanisms that control gene expression are not fully understood yet. In particular, a lot of methods exist to elucidate TF or miRNA-related regulatory networks, but comparable information to explicitly connect them is still lacking. Given a transcriptional and a post-transcriptional regulatory network, different possible ways to connect them are in principle possible: recently, a strong focus regarding the study of local mixed interactions has emerged in several works [4-10].

**Figure 1:**
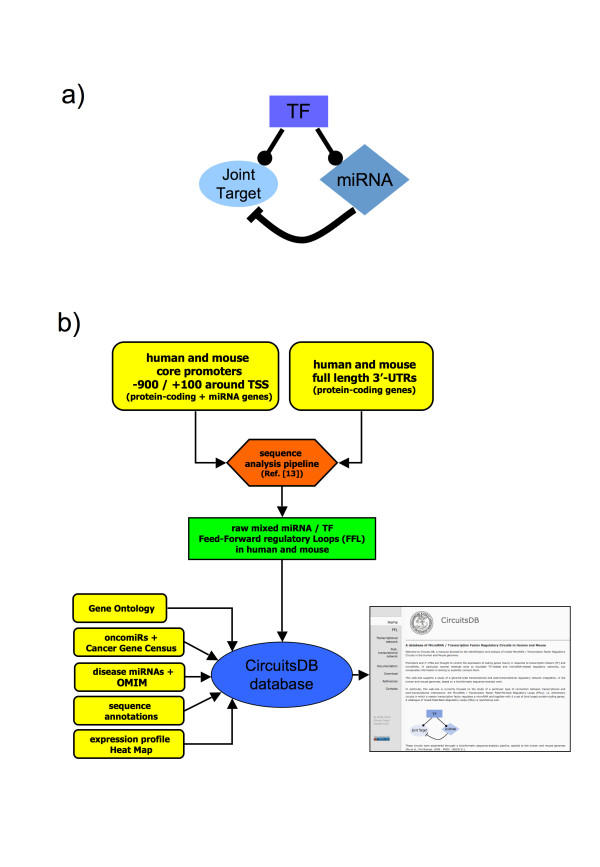
**Mixed miRNA/TF Feed-Forward Loops and **CircuitsDB** construction pipeline**. a) Representation of a typical mixed Feed-Forward regulatory Loop (FFL) included in CircuitsDB. Circuits are composed by a master Transcription Factor (TF, square box) that regulates a microRNA (miRNA, diamond-shaped box) and together with it a Joint Target protein-coding gene (round box). Inside each circuit, -• indicates transcriptional activation/repression, whilst ⊣ post-transcriptional repression. b) Flow-chart of the strategy used for the construction of CircuitsDB. We first built a genome-wide catalogue of putative core promoter regions for protein-coding and miRNA genes, plus a set of 3'-UTRs for protein-coding genes, in human and mouse. Then we used the analysis pipeline developed in our previous work [13] to infer a dataset of mixed regulatory FFLs, in human and mouse. Finally we integrated different kinds of biological annotations to support the circuits' properties; the CircuitsDB web-site allows a dynamic exploration of such properties.

In this respect, it is important to notice that the transcription of miRNAs is widely regulated by POLII type promoters [11], and that co-expressed miRNAs are found to be regulated by common TFs [12]. Stemming from these considerations, we previously developed a computational framework for the study of connections between transcriptional and post-transcriptional (miRNA-mediated) regulatory interactions in the human genome [13]. We concentrated our attention on a particular class of local regulatory circuits (i.e. *network motifs*) in which a TF regulates an miRNA and together with it a set of Joint Target protein-coding genes. These circuits, called mixed miRNA/TF Feed-Forward regulatory Loops (FFLs, Figure 1a), were identified through a bioinformatic pipeline, mainly based on an ab-initio sequence analysis of human and mouse genomes. Once equipped with the catalogue of FFLs, we studied different ways to characterize their biological behaviors and implications. In particular, data were used to investigate connections between the mixed regulatory circuits involved in cancer.

Here, we present CircuitsDB, a user-friendly web-server that includes and extends our previous work [13].

In addition to the human catalogue of mixed FFLs, the database now contains full datasets relative to the mouse genome as well. Data are stored in a relational database that can be accessed through a dynamic web interface. The interface is composed by series of interactive on-line forms that allow the users to start with their favourite TF or miRNA or gene, and to follow their mutual connections in regulatory loops. Sequence information as well as functional data are provided (Gene Ontology, genomic annotations and positions of the putative regulatory sites, links to cancer or, more in general, disease information, patterns of tissue expression). Finally, a Wiki is also present, so that users can give their own feedback regarding the proposed interactions.

CircuitsDB is freely available at: http://biocluster.di.unito.it/circuits/.

## Construction and content

CircuitsDB was constructed using a bioinformatic pipeline mainly based on an ab-initio sequence analysis applied to regulatory regions of human and mouse genomes. In this section, we first describe the dataset of genomic regions used for the definition of our database. Second, we summarize the methodology originally used in [13] for the identification of the transcriptional and post-transcriptional players of the regulatory networks, in human and mouse and the approach used for their integration in mixed Feed-Forward regulatory Loops (FFLs). Finally, we describe the content and the structure of our web-accessible database.

### Definition of the dataset of genes and regulatory regions used to infer the transcriptional and post-transcriptional networks

The promoter regions for protein-coding and microRNA (miRNA) genes as well the 3'-UTRs were defined according to [13]. Gene definitions, sequences, and functional annotations were extracted from the Ensembl database [14], release 46 and from miRBase, version 9.2 [15]. The promoter region we selected for protein-coding genes corresponded to (-900/+100) nts around the Transcription Start Site (TSS), being the TSS at position +1. For each protein-coding gene, if more than one transcript was present, we used only the longest one. For miRNA genes, we first grouped pre-miRNAs in the so called Transcriptional Units (TUs) [16] and associated the promoter of the most 5'-upstream member to all the pre-miRNAs belonging to the TU itself. Then, based on the fact that the pre-miRNAs were inter- or intra-genic, we defined the following promoters. For inter-genic pre-miRNAs the promoter corresponded to (-900/+100) nts upstream of the TSS of the first pre-miRNA in the TU. The same was true for intra-genic pre-miRNAs which showed opposite orientation with respect to the hosting protein-coding gene. Finally, if the pre-miRNAs were intra-genic but sharing the same orientation of the hosting protein-coding gene, the promoter region was considered coincident with the one defined for the protein-coding host gene.

This procedure was implemented here for both human and mouse genomes. For subsequent analysis we considered only protein-coding and pre-miRNA genes showing at least a direct one-to-one orthology between the two genomes (from [14] and [16]). The final dataset of promoter regions is composed of a collection of 21446 (21316 protein-coding plus 130 pre-miRNA) human and 21944 (21814 protein-coding plus 130 pre-miRNA) mouse regulatory sequences. The 130 pre-miRNAs included in our work encode for 193 mature miRNAs (see Supplementary File S1 of [13]). For protein-coding genes, we then downloaded the 3'-UTR regions, considering only the longest transcript in case of multiple alternative isoforms. We ended up with only 17486 human and 15921 mouse sequences, since not all the genes have a well defined 3'-UTR in the Ensembl database. All the sequences were Repeat-Masked using Ensembl default parameters.

### Oligo analysis and definition of mixed microRNA/Transcription Factor regulatory Feed-Forward Loops

Details about the oligo analysis are listed in [13]; here we report our main choices and results (Figure 1b). Briefly, we scanned all the promoter regions and the 3'-UTRs for conserved-overrepresented oligos (6 to 9 nts for promoters; 7 nts for the 3'-UTRs) with potential regulatory roles (as Transcription Factor Binding Sites, TFBS, for promoters or miRNA seeds for the 3'-UTRs). By doing so, we fixed 0.1 as False Discovery Rate (FDR) in the oligo analysis pipeline. To assess the oligos surviving the motif-finding analysis, we used a catalogue of known TFBS consensus from the Transfac database [17] and from [18] for the oligos located in promoter regions, manually filtering out those TFs characterized by very long or too degenerate consensus sequences. Similarly, we used a catalogue of known miRNA seeds derived from the mature miRNAs included in our study to identify significant oligos located in the 3'-UTRs (see [19] and [20] for additional details concerning the used algorithms). The above analysis was performed here in parallel for human and mouse. In human, for the transcriptional network, we obtained a catalogue of 2031 significant oligos that could be associated to known TFBSs for a total of 115 different TFs. These 2031 oligos targeted 21399 genes (21219 protein-coding and 180 mature miRNAs). For the post-transcriptional network, we ended up with a library of 182 significant oligos, each of them matching with at least one seed present in 140 out of our 193 mature miRNAs and targeting a total of 17266 protein-coding genes. We obtained rather similar results for the mouse: the transcriptional network is composed of 22054 genes (21875 protein-coding and 179 pre-miRNAs) and 115 different TFs targeting the 2031 significant oligos. On the other hand, the post-transcriptional network includes 15755 genes, targeted by 178 significant oligos corresponding to 143 mature miRNAs.

Once we obtained these two regulatory networks, we focused on the integration of the two datasets in order to construct a catalogue of mixed miRNA/TF FFLs (Figure 1a). In human, that integration included 5030 different "single target circuits", each of them defined by a single TF as master regulator, a single mature miRNA and a single protein-coding Joint Target. From these single target circuits we constructed "merged circuits" grouping together the FFLs sharing the same TF and the same miRNA, thus obtaining 638 merged circuits. These circuits involved a total of 101 TFs, 133 mature miRNAs and 2625 Joint Target genes. In mouse, we found 6684 different "single target circuits", which could be grouped in 850 "merged circuits", involving a total of 94 TFs, 142 mature miRNAs and 2968 Joint Target genes. 30 single target circuits were conserved between human and mouse: they share the TF, the miRNA as well as the Joint Target being one-to-one orthologs.

The prediction of reliable miRNA-mediated post-transcriptional regulatory interactions is still an open issue in computational biology and it is well known that different approaches can lead to very different outcomes [21]. To address this problem, we included in CircuitsDB conserved miRNA-target predictions obtained from two external resources, namely TargetScan [22] and TargetMiner [23]. We dowloaded from the TargetScan website http://www.targetscan.org/ inferred miRNA targets for the human and mouse genomes and we mapped Entrez Gene symbols and miRNA family names provided on the Ensembl stable identifiers (ids) for protein-coding genes and pre-miRNAs present in our database of FFLs. A similar procedure was implemented for the human genome-wide predictions obtained from the TargetMiner website http://www.isical.ac.in/~bioinfo_miu/, where human RefSeq ids were again mapped on Ensembl gene stable ids. By TargetScan or TargetMiner we could confirm 1434 out of the 5030 human FFLs included in our study at the post-transcriptional level. Similar results were obtained also for the murine case, resulting in 1107 FFLs having the post-transcriptional link confirmed by TargetScan. These data are reported in Additional File 1 (for human) and Additional File 2 (for mouse).

Furthermore, we also investigated whether in our database connections between TF and miRNAs were present in mixed Feed-Back regulatory Loops (FBLs), i.e. situations in which a master TF regulates an miRNA, being itself the target of the regulated miRNA at the post-transcriptional level. To this end, in order to recognize the miRNA - TF post-transcriptional interactions, we manually prepared a translation table in which Transfac TF ids were associated to known Ensembl gene stable ids, where possible. We ended up with a catalogue of 113 mixed FBLs in human and 38 FBLs in mouse, having the post-transcriptional link confirmed by at least one of the supporting databases included.

### Fuctional annotations, cancer and disease genes

Once equipped with the catalogue of mixed miRNA/TF Feed-Forward loops, we investigated their functional properties with several different criteria with a focus on functional annotations according to the Gene Ontology (GO) database [24] and to their relevance in cancer or other diseases. In the present version of CircuitsDB, these three types of biological annotations are included in the on-line web-service.

Gene product GO annotations for the TF and Joint Target protein-coding genes were downloaded from the Ensembl database, version 46.

Regarding the identification of cancer related genes, we obtained a list of oncomiRs from [25,26] and [27] while for the protein-coding target genes we enumerated a list of genes showing mutations in cancer based on the Cancer Gene Census catalogue [28]. We then focused on the annotation of CircuitsDB genes and miRNAs in terms of genetic diseases: for protein-coding genes we used the established OMIM[29] catalogue, whilst the HMDD miRNA-disease database [30] was interrogated to annotate the miRNAs present in our FFLs.

### Comparison with experimentally supported regulatory interactions for each of the circuit's link

We then compared our predicted human miRNA-target links (miRNA ⊣Joint Target) with two databases of experimentally supported information, Tarbase [31] and the component of miRecords [32] reporting validated miRNA-target links. 140 miRNAs, used in our post-transcriptional network, were also present in Tarbase or miRecords. On average, 11.5% of our predicted miRNA targets were already validated experimentally. It was of interest to compare the other two computational algorithms for miRNA target predictions, TargetScan and TargetMiner, with our own algorithm, in the same benchmarking setting, in order to compare the relative performances. To this end, we applied a binomial test to assess the proportions of experimentally supported predictions (implemented as the function prop.test() in the R [33] statistical environment); for the sake of the comparison, this test was applied separately to each miRNA in common between our post-transcriptional network and TargetScan (33) or TargetMiner (30). Setting the confidence threshold to 0.01, no significant dierence was found for 30 out of 33 miRNAs, when using TargetScan, and for 29 out of 30 miRNAs, when using TargetMiner. Therefore, we can conclude that the proportions of true positives recognized separately by each algorithm, evaluated on the miRNA pool present in our database, are substantially comparable (see Additional File 3).

Eventually, we also assessed the reliability of our predictions separately for the other two types of links in a circuit: the TF -• Joint Target and the TF -• miRNA regulatory interactions.

A proof of principle of the reliability of the TF -• Joint Target links was already established in our previous work [13], where we compared our predictions with the experimentally validated results reported in [34], a large-scale study of direct MYC binding target genes in a model of human B lymphoid tumors performed by chromatin immunoprecipitation coupled with pair-end ditag sequencing analysis (ChIP-PET). The intersection between [34] and our predicted MYC interactions proved to be statistically significant (*p *= 1.1 × 10^-6^, Fisher's test).

We then proceeded to compare our predicted TF -• miRNA regulatory interactions links with TransmiR [11], a literature-based database of TF - miRNA links. At present, we were able to safely identify only 16 TF names in common between our dataset and the TransmiR database. 5 TF - miRNA links were found in common out of the 36 indentified by TransmiR, involving a transcription factor and an miRNA present in our dataset.

### Tissue expression Heat Map

Due to the sequence analysis pipeline that we adopted to identify the FFLs in CircuitsDB, we were not able to recognize if the action of the master TF was activating or repressing its targets and thus if the FFL that we obtained was of the so-called Type I or Type II [5,13]. Moreover, not only the two types of circuits may lead to very different behaviours in terms of expression patterns of their components [5], but also the understanding of the consequences of miRNA-target interaction is currently challenged [35-37].

Therefore, we decided to simply give the users the possibility to explore the expression values across several tissues for the TF, miRNA and Joint Target belonging to a given FFL by means of a graphical heat map representation. For this purpose, we collected the expression profiling data for 175 miRNAs over 24 human organs from [38] and for more than 40000 human protein-coding transcripts as microarray probe sets over 79 human tissues from [39]. We were able to identify 14 tissue types in common between the two datasets: AdrenalGland, BoneMarrow, Brain, Heart, Kidney, Liver, Lung, Lymph, Pancreas, Placenta, Prostate, Testis, Thymus, Uterus (see also [23]). For the protein-coding genes, we then used the BioMart tool of the Ensembl database to map the original probe sets on the Ensembl gene stable ids used in our CircuitsDB. If more than one probe matched on the same Ensembl id, we retained as expression values for that gene the mean values, for each tissue type. For both miRNAs and protein-coding genes the expression values were then *log*2-transformed and the *Z *- *score *with respect to the mean over all the tissues was evaluated. These values were finally used to create, for each mixed FFL, a heat map, composed by 14 rows (corresponding to the 14 different tissues) and 3 columns, corresponding to the TF, miRNA and Joint Target embedded in the FFL.

## Utility

**Figure 2:**
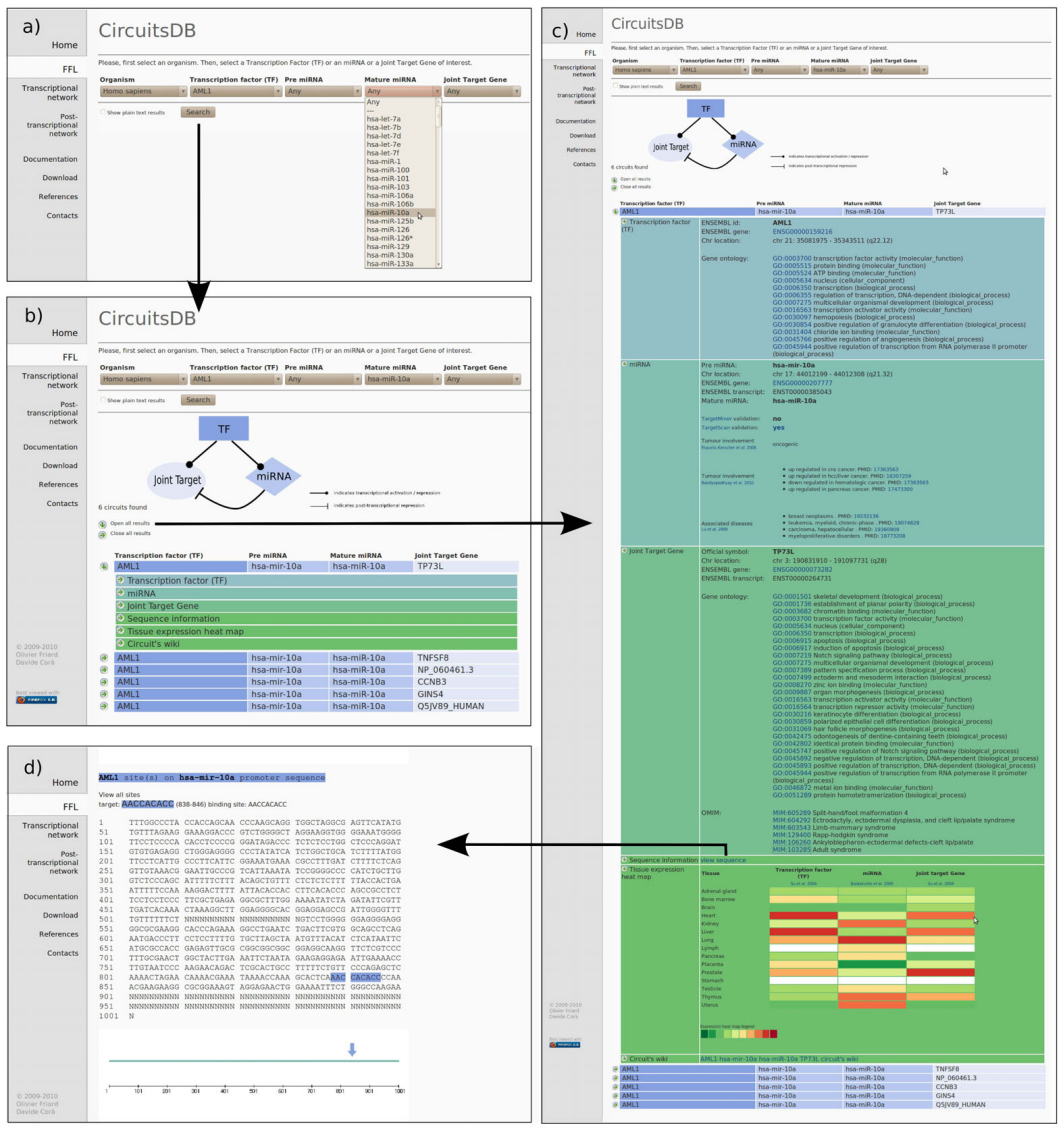
**Example of **CircuitsDB** usage**. Graphical snapshots of a typical working session with CircuitsDB. a) In the first step the user selects an organism of interest and at least one search key among the available ones: Transcription Factor (TF) or microRNA (miRNA) or Joint Target gene name. b) In the second panel a list of mixed TF/miRNA regulatory circuits is retrieved. c) In the third panel an example of CircuitsDB output is shown: a single mixed Feed-Forward Loop is identified and its components are reported in a tabular-like fashion. The user can explore TF additional information, as well as miRNA and Joint Target annotations. A sequence analysis panel and a Wiki for community feedback are available. d) Example of sequence analysis panel: TF binding sites are explicitly highlighted on the miRNA core promoter region.

CircuitsDB was built in the PHP script language and as a MySQL relational database system on a Linux server. In the MySQL database pre-compiled transcriptional and post-transcriptional networks, the dataset of mixed FFLs and all fuctional and biological information, for both human and mouse, are stored. The interactive web interface allows the user to first select an organism of interest, then select a TF, an miRNA id or a protein-coding gene name (or a combination of these three elements) and query the database in order to retrive a catalogue of mixed miRNA/TF Feed-Forward regulatory Loops in which the search keys are involved (Figure 2a and 2b).

Query's results are displayed in a synthetic tabular-like view, with single target circuits on separate lines. Different color codes for the different circuit elements are used. The view relative to each single-target circuit can be expanded into six related sub-panels showing additional detailed information, similarly to the graphical strategy adopted e.g. in [31].

The additional information is divided into six categories: *Transcription Factor (TF), miRNA, Joint Target gene, Sequence, Tissue Expression Heat Map and Circuit's Wiki *(Figure 2c). The TF information category gathers mostly biological properties of the master TF regulating the circuits: its Ensembl annotations, chromosome location and Gene Ontology annotation, if available. The miRNA information category is composed of the Ensembl annotations of the pre-miRNA composing the circuit, its chromosome location and corresponding mature miRNA ids. Tumor involvement fields provide annotations of miRNAs known to be involved in cancer. In parallel to that, a disease field reports whether the miRNA is connected to a known disease, if any. The Joint Target gene information panel contains again Ensembl annotation for the protein-coding Joint Target gene, the gene official name as well as GO annotations. Again, a tumor involvement field shows possible links with cancer. The OMIM fields displays the corresponding mendelian disease associated to the current gene. With the Sequence information link the user is redirected to an additional web-page in which the promoter sequence of the miRNA or the promoter and 3'-UTR of the Joint Target present in the circuit are explicitly displayed. TFBS identified with our algorithm and miRNA binding sites are highlighted on the sequences, thus providing direct information for experimental tests (Figure 2d). In the Tissue Expression Heat Map viewer we interactively generated a heat map of tissue specificity for the TF, miRNA and Joint Target belonging to the circuit. Furthermore, we provide CircuitsDB with an embedded Wiki system, that allows interested users to share their knowledge and efforts in annotating circuits: the last category, *Circuit's Wiki*, redirects the user to editable web pages, where a registered user can add personal information in addition to the biological annotations already included in our database.

A Download section provides all the CircuitsDB content as flat files. A Reference section reports a list of the most important data sources used for the construction of our database.

Finally, two additional sections of the database are directly accessible through links in the home page: "Transcriptional" and "Post-Transcriptional Network". In the first one, the user can explore the transcriptional network used for the development of the mixed FFLs catalogue, in human and mouse. Entry points can be a TF of interest, a gene or a DNA oligo. It is worth mentioning that this section allows direct inspection of the subset of our transcriptional regulatory networks involving miRNAs as targets, i.e. the collection of putative TFBS identified on promoter regions for miRNA genes. In the second one, users can explore our post-transcriptional networks, for human and mouse. Entry points can be an miRNA of interest, a gene or again a DNA oligo.

## Discussion

CircuitsDB is a database where transcriptional and post-transcriptional (miRNA mediated) network information is fused together in order to propose and recognize non trivial regulatory combinations. Figure 2 shows the main steps that a user should follow during the investigation of the proposed circuits in CircuitsDB web-site: identification of a FFL according to a TF, miRNA or Joint Target gene id (Figure 2a and 2b); exploration of the circuit components and related annotations (Figure 2c); investigation of the regulatory sites comprised in the circuit corresponding to the identified sequences (Figure 2d).

In [13] several examples in which mixed Feed-Forward Loops could exert synergistic biological effects have already been discussed (the MYC/hsa-miR-20a;miR-17-5p circuit, the AREB6/hsa-miR-375 circuit, the MEF-2/hsa-miR-133a circuit, the C-REL/hsa-mir-199a circuit and the HSF2/hsa-let-7f circuit). Here, we will discuss other examples of single-target circuits potentially linked to cancer that we obtained from CircuitsDB.

One circuit involves the master transcription factor Runx1 or Acute Myeloid Leukemia 1 (AML1), miR-10a and the p63 (TP73L), three genes found implicated in leukemia. AML1 is the target of multiple chromosomal translocations in human leukemia and the TEL-AML1 oncogene is the hallmark translocation in Childhood Acute Lymphoblastic Leukemia [40]. miR-10a was proven to be overexpressed and functionally relevant in various tumors, including AML [41] while p63 is a critical transcriptional regulator of cancer cells [42]. This FFL might also coordinate the physiological hematopoiesis during embryonic development since Runx1 is essential for the generation of definitive haematopoietic cells from haemogenic endothelium as shown using a conditional deletion of Runx1 [43]. Nevertheless, p63 has been found to be relevant for tissue development [44,45] and miR-10a is one of the most upregulated miRNA during endodermal differentiation from human embryonic stem cells [46].

Another potentially relevant circuit for tumorigenesis, in particular for ovarian cancer, a leading cause of death from gynecologic malignancies, is composed of HoxA4, miR-125b and ERBB3. HOX genes are transcription factors that control morphogenesis, organogenesis as well as differentiation and that play an important role in ovarian cancer progression by controlling cell migration [47]. miR-125b has been found to be differentially expressed in serous ovarian carcinomas compared with normal ovarian tissues [48]. At the same time, ERBB3 is a tyrosine kinase receptor often activated in ovarian cancer and perturbation of ERBB3-dependent signal transduction by RNA interference resulted in decrease disease progression and prolonged survival in murine models, identifying ERBB3 as a potential therapeutic target in ovarian cancer [49]. The identification of an miRNA, i.e. miR-125b, that could downregulate ERBB3 would be very valuable for ovarian cancer treatments.

Another interesting association with cancer can be observed in the SOX-5/miR-29a/SPARC circuit. Decreased expression of SPARC, an important mediator of cell-matrix interaction, was previously observed in Nasopharyngeal carcinoma (NPC) and in the same system SOX-5 turned out to be upregulated [50]. Considering that SOX-5 overexpression in NPC tumors correlates clinically with poor survival it is essential to understand how SOX-5 regulates tumor progression. It is conceivable that that SOX-5 down-regulates SPARC expression directly at the level of transcription, while regulating positivey miR-29a transcription: this would result in coordinate downregulation of SPARC at the post-transcriptional level. Considering that several evidences connect miR-29a with epithelial tumor invasion and metastasis formation as well as epithelial-mesenchymal transition (EMT) [51] miR-29a could represent a main regulation of SPARC and experimental validations should be carried out.

Some mixed circuits have already been studied from an experimental point of view. For instance, in [52] the authors investigated the interaction between the miR-17-92 cluster, the Myc oncogene and the E2F1 transcription factor, being E2F1 an additional target of Myc that promotes cell-cycle progression (this circuit is predicted also from our analysis and thus present in CircuitsDB). In [53] Brosh and colleagues analyzed mixed FFLs in the framework of the mammalian cell proliferation control network. They again concentrated on a network architecture that includes the transcription factor E2F1 and a family of 15 miRNAs, which co-regulate mutual target genes transcriptionally and post-transcriptionally and whose cooperative action reinforces cellular proliferation. Then, this FFL appears to be repressed by p53, possibly by promoting senescence and suppressing cancer progression.

Although in our work we focused on the circuits' properties in relation to cancer biology, by means of the already established biological features of their components, other possible functions might exist for the proposed catalogue of mixed FFLs. In particular, in the original formulation of [4], the wording for mixed circuits referred to an evolutionary perspective: looking at how "canalizing genes", essential for higher organism development, could be influenced by miRNA regulatory networks that act as stabilizers for noise fluctuations in gene expression (type I or incoherent FFLs) [35] and [36] seem to shed new light in supporting this alternative hypotesis for FFLs functioning and in [54] was recently proposed, through stochastic modeling and simulations, a mathematical model for that. It is worth noting that in parallel to this, mixed regulatory circuits are also emerging as key players in regulatory networks of Embryonic Stem Cells (ESC) [55]. Moreover, the interplay between TFs, miRNAs and shared targets is able to influence ESC differentiation and act as defining factors in Induced Pluripotent (iPS) and Cancer Stem Cells (CSC) [56].

## Conclusions

We present CircuitsDB, a public web application devoted to the study of interactions between transcriptional and post-transcriptional regulatory interactions. CircuitsDB is currently mainly focused on the study of mixed miRNA/TF Feed-Forward Loops, i.e. regulatory circuits in which a master TF regulates an miRNA and, together with it, a set of Joint Target protein-coding genes. These circuits were assembled in our previuos experience [13] in the human case, based on a bioinformatic ab-initio analysis. Here, we expanded to the murine case our dataset and provided an integrated web-service to explore and directly investigate such relationships in terms of their sequence and several types of functional annotations. A catalogue of mixed mixed miRNA/TF Feed-Back Loops is also presented.

We consider CircuitsDB only the first step of more advanced studies. In particular, we plan to further extend our work to include additional types of mixed miRNA/TF local interactions and other types of post-transcriptional regulators. Prediction methods to infer TF and miRNA regulatory networks also continue to evolve and genome-wide experimental dataset of TF and miRNA interactions will be available in the near future. In subsequent releases, additional bioinformatic methodologies and experimental data for the construction of the database could be easily incorporated in our server.

## Availability and requirements

The CircuitsDB web-service is freely available at http://biocluster.di.unito.it/circuits/. Detailed documentation can be accessed by a link on the left bar in the home page and includes various explanatory applications.

## Abbreviations

TFs: Transcription Factors; miRNAS: microRNAs; FFLs: Feed-Forward regulatory Loops; FBLs: Feed-Back regulatory Loops.

## Authors' contributions

OF wrote the software implementation of CircuitsDB. AR and DT participated in data analysis and in their biological assessment. MDB provided funding and resources for the project. DC designed, coordinated the project and partecipated in data analysis. DC wrote the paper and all the authors read and approved the final version.

## Supplementary Material

Additional file 1 **Catalogue of human mixed Feed-Forward regulatory Loops included in **CircuitsDB. The complete list of mixed Feed-Forward regulatory Loops (FFLs) included in the current release of CircuitsDB is reported, for the human genome. Each line corresponds to a single-target closed FFL. The first column includes the FFL id, composed by the Transcription Factor (TF) name and miRNA gene ids (Ensembl stable identifier, pre-miRNA and mature miRNA ids according to the standard nomeclature of miRBase). The second column shows the Joint Target protein-coding gene (Ensembl stable identifier and HGNC standard id). The third and fourth column report the validation of the miRNA ⊣ Joint Target post-transcriptional interaction according to the TargetScan and TargetMiner databases, respectively. Click here for file

Additional file 2 **Catalogue of mouse mixed Feed-Forward regulatory Loops included in **CircuitsDB. The complete list of mixed Feed-Forward regulatory Loops (FFLs) included in the current release of CircuitsDB is reported, for the mouse genome. Each line corresponds to a single-target closed FFL. The first column includes the FFL id, composed by the Transcription Factor (TF) name and miRNA gene ids (Ensembl stable identifier, pre-miRNA and mature miRNA ids according to the standard nomeclature of miRBase). The second column shows the Joint Target protein-coding gene (Ensembl stable identifier and Mouse gene symbol). The third column reports the validation of the miRNA ⊣ Joint Target post-transcriptional interaction according to the TargetScan database. Click here for file

Additional file 3 **Results of the comparison with experimentally validated miRNA-gene pairs**. The table shows the results of the binomial test used to compare the proportions of experimentally supported miRNA-gene interactions between CircuitsDB and TargetScan or TargetMiner. Only miRNAs present in at least one circuit, included by TargetScan or TargetMiner and with a minimum of one validated target were used (0.01 significance threshold). Click here for file
