# Supplementary material for: CircuitsDB: a database of mixed microRNA/transcription factor feed-forward regulatory circuits in human and mouse
Source: BMC Bioinformatics. 2010 Aug 23;11:435. doi: 10.1186/1471-2105-11-435 (PMC2936401; doi:10.1186/1471-2105-11-435)
Supplement: Additional file 3 — Results of the comparison with experimentally validated miRNA-gene pairs. The table shows the results of the binomial test used to compare the proportions of experimentally supported miRNA-gene interactions between CircuitsDB and TargetScan or TargetMiner. Only miRNAs present in at least one circuit, included by TargetScan or TargetMiner and with a minimum of one validated target were used (0.01 significance threshold). [file 1471-2105-11-435-S3.PDF]

The table shows the results of the binomial test to compare the proportions of experimentally supported predictions between CircuitsDB and TargetScan or TargetMiner (by means of the function prop.test() in the R statistical environment). Only miRNAs present in at least one circuit, included by TargetScan or TargetMiner and with at least one validated target were used. MicroRNAs non testable by TargetScan or TargetMiner are marked by 'na'. The significance threshold is set to 0.01. The columns report: the mature microRNA MirBase identifier, the number of target genes according to Tarbase, the number of target genes common to CircuitsDB and Tarbase, the number of target genes according to CircuitsDB, the number of target genes common to TargetScan (or TargetMiner) and Tarbase, the number of target genes according to TargetScan (or TargetMiner), the P value of the test.

microRNA	Tarbase	CircuitsDB		TargetScan		P value	TargetMiner		P value
	no. calls	no. true calls	no. calls	no. true calls	no. calls		no. true calls	no. calls	
hsa-miR-15a	64	2	57	8	1168	0.119	16	2354	0.094
hsa-miR-106a	7	1	384	6	1114	0.798	6	2841	1.000
hsa-miR-200a	2	2	205	1	580	0.345	1	104	1.000
hsa-miR-29a	4	1	215	3	964	1.000	2	1075	1.000
hsa-miR-141	5	1	205	2	580	1.000	na	na	na
hsa-let-7g	3	1	527	2	907	1.000	1	1440	1.000
hsa-miR-372	3	1	492	2	663	1.000	3	2113	1.000
hsa-let-7a	7	1	527	4	907	0.754	4	1440	1.000
hsa-miR-93	3	2	492	3	1114	1.000	3	2841	0.336
hsa-miR-155	95	7	423	21	303	0.001	24	913	0.366
hsa-miR-200b	3	2	205	2	894	0.332	3	274	1.000
hsa-miR-20a	4	2	436	4	1114	1.000	4	2841	0.399
hsa-miR-99b	1	1	220	1	41	0.717	na	na	na
hsa-miR-1	185	11	307	68	639	0.000	48	1131	0.722
hsa-miR-373	70	11	464	17	663	0.991	23	2113	0.049
hsa-miR-21	25	3	139	4	224	1.000	7	885	0.289
hsa-let-7e	3	1	428	1	907	1.000	1	1439	0.944
hsa-miR-200c	2	2	205	2	894	0.332	3	274	1.000
hsa-miR-26a	5	1	193	3	729	1.000	3	1767	0.859
hsa-miR-130a	6	3	361	4	802	0.789	4	2420	0.073
hsa-miR-101	5	2	262	2	687	0.657	1	1336	0.116
hsa-miR-16	155	7	57	35	1168	0.001	52	2354	9.46E-06
hsa-miR-29c	14	7	215	11	964	0.048	9	1075	0.010
hsa-miR-125b	4	2	347	1	644	0.586	1	488	0.766
hsa-miR-24	7	1	209	1	467	1.000	2	941	1.000
hsa-miR-96	3	2	289	2	866	0.564	2	1188	0.365
hsa-miR-206	6	2	307	1	639	0.516	2	1131	0.430
hsa-miR-106b	4	2	436	3	1114	0.926	3	2841	0.271
hsa-miR-99a	1	1	220	1	41	0.717	na	na	na
hsa-let-7b	82	7	527	24	907	0.143	23	1440	0.823
hsa-miR-182	2	2	140	2	927	0.148	2	1585	0.031
hsa-miR-20b	5	1	476	4	1114	1.000	5	2841	1.000
hsa-miR-29b	8	1	215	5	964	1.000	2	1075	1.000
